# Memristive and Tunneling Effects in 3D Interconnected Silver Nanowires

**DOI:** 10.1021/acsomega.2c07171

**Published:** 2023-02-07

**Authors:** Chloé Chopin, Simon de Wergifosse, Nicolas Marchal, Pascal Van Velthem, Luc Piraux, Flavio Abreu Araujo

**Affiliations:** Institute of Condensed Matter and Nanosciences, Université catholique de Louvain, Place Croix du Sud 1, 1348 Louvain-la-Neuve, Belgium

## Abstract

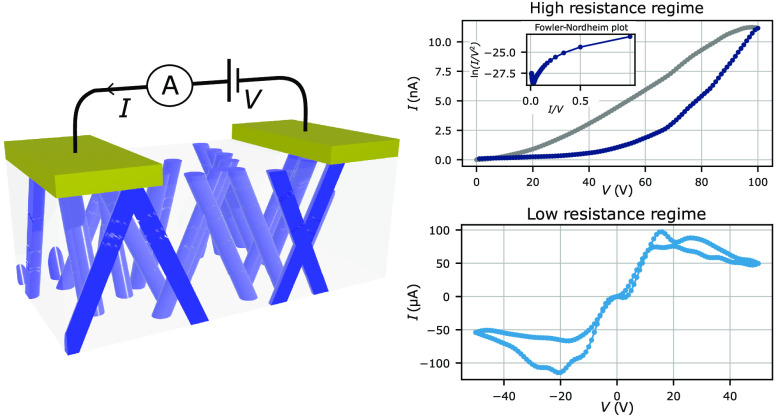

A network of silver nanowires (Ag-NWs) is grown by electrodeposition in a nanoporous membrane with interconnected nanopores. This bottom-up approach fabrication method gives a conducting network with a 3D architecture and a high density of Ag-NWs. The network is then functionalized during the etching process, which leads to a high initial resistance as well as memristive behavior. The latter is expected to arise from the creation and the destruction of conducting silver filaments in the functionalized Ag-NW network. Moreover, after several cycles of measurement, the resistance of the network switches from a high-resistance regime in the GΩ range with tunnel conduction to a low-resistance regime presenting negative differential resistance in the kΩ range.

## 1 Introduction

Memristors were theorized by Chua in 1971 and are described as the missing component linking charge and flux.^1^ In 2008, Strukov et al.^2^ showed that some nanodevices exhibiting a nonlinear hysteretic *I*–*V* curve are in fact memristive devices. This phenomenon was previously observed in memory based on resistive switching.^3^ Resistive switching arises from different phenomena,^3,4^ including the formation and destruction of conductive filaments (CFs) from the migration of metallic ions of Cu or Ag, for example.^5,6^ The formation of CF arises in a variety of structures, like atomic switches, which can be integrated in a crossbar structure,^6^ or nanowire (NW) networks,^7^ where more than one interconnection between NWs can be achieved. Several materials^7^ can be used to fabricate the NWs, including Cu, Ni, Ti, or Ag. NWs composed of silver can be used for transparent electronics^8^ and have a low electrical resistivity.^9^

Several fabrication processes are used to fabricate Ag-NW networks, like growing Ag-NWs from Cu seeds^10^ or with a polyol synthesis^11^ and a polyvinylpyrrolidone (PVP) coating^12−15^ (more fabrication processes are detailed in this review^7^). PVP coated Ag-NWs can exhibit different densities of Ag-NWs in the sample^14^ as well as a larger resistance because of the PVP coating.^12^ Several post-treatments can be used to increase the connections between Ag-NWs, including thermal annealing, mechanical pressing, and other techniques.^9,12^ On the contrary, one can use post-treatments to increase the resistance of the network and create opportunities for the creation of Ag CFs so that the network exhibits memristive behavior. It can be done either thanks to the encapsulation of the Ag NWs in an insulating shell like PVP,^12,14−16^ where conductive filaments grow across the PVP insulating layer,^15^ or by fragmenting the Ag-NWs with UV/ozone irradiation, followed by annealing.^13^

In this work, we propose a memristive device made of three-dimensional (3D) interconnected Ag-NWs. Those are deposited inside a nanoporous membrane, which allows a bottom-up fabrication of a random but ordered NW network with a high number of interconnections.^17^ The Ag-NWs are then weakened during the etching step in order to have memristive properties.

## 2 Experimental Section

### 2.1 Electrochemical Deposition

**Figure 1:**
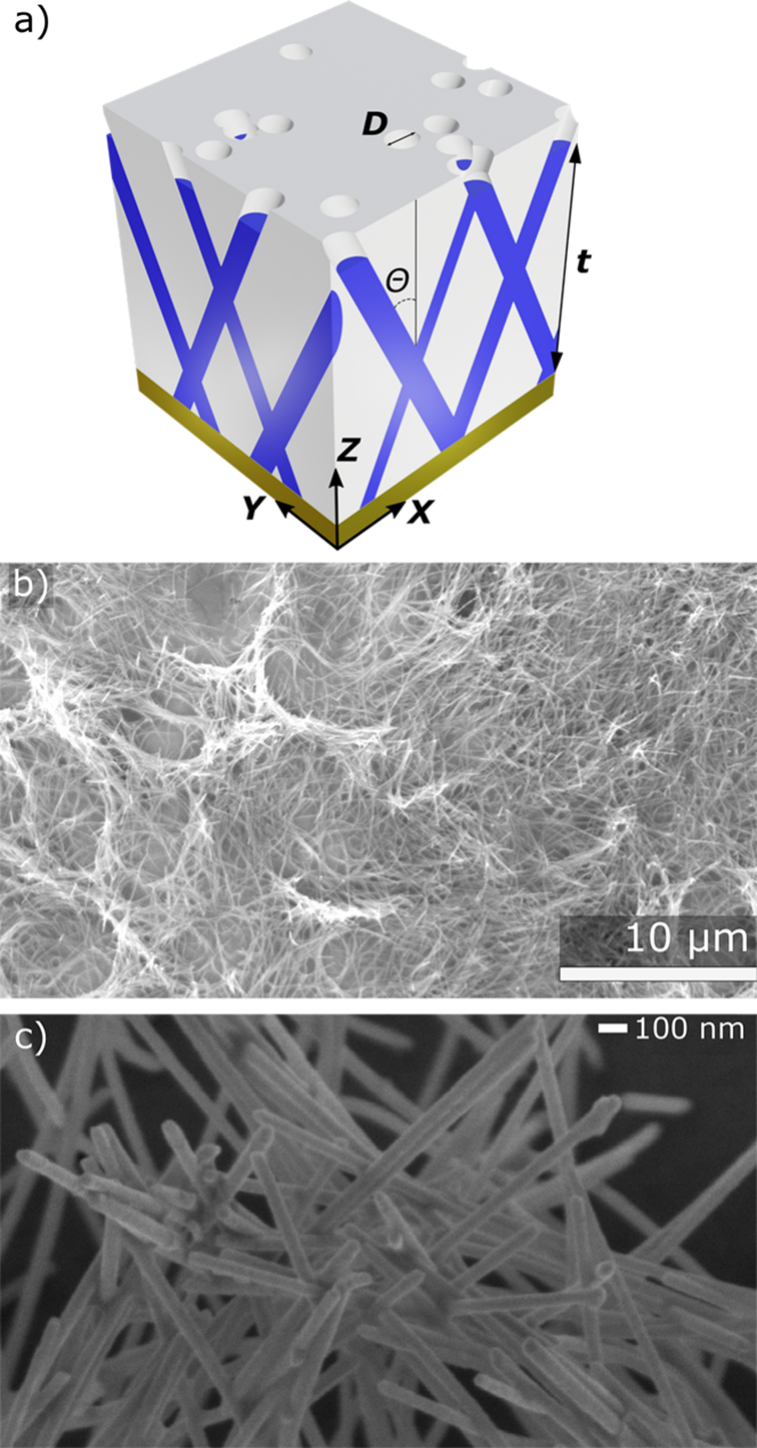
(a) Scheme of the 3D interconnected Ag-NW network inside the membrane. The nanowires are represented in blue with *t* as the network thickness, *D* as the nanowire diameter, and θ as the angle with the normal. Different types of interconnections are shown on the edges. (b) SEM image of the network after the dissolution of the membrane. The network collapses because of the low porosity and the thin diameter of the Ag-NWs. (c) Zoomed-in view of the NW intersections.

Electrochemical deposition in an ion track nanoporous polycarbonate (PC) membrane was employed to produce 3D interconnected Ag-NWs, as shown schematically in Figure 1a. The nanoporous PC membrane with interconnected nanopores was fabricated by exposing a 25 μm thick PC film to a two-step irradiation process. The topology of the membrane was defined by exposing the film to a first irradiation step at two fixed angles of −25° and +25° with respect to the normal axis of the film plane. In practice, the angles are between 20° and 25°. After the PC film was rotated in the plane by 90°, the second irradiation step took place at the same fixed angular irradiation flux to finally form a 3D nanoporous network. The diameter of the latent tracks was enlarged by following a previously reported protocol^18^ to obtain a membrane with an average pore diameter of 30 nm and a volumetric porosity of about 0.4%. Next, the PC templates were coated on one side using an e-beam evaporator with a metallic Cr (3 nm)/Au (250 nm) bilayer to serve as a cathode during the electrochemical deposition. The Ag-NWs were fabricated by electrodeposition using a silver cyanide-based commercial electrolyte (Silver-Bright-100, Metakem GmbH) in a two-electrode configuration at room temperature by applying a constant potential of −1.5 V versus a double-junction Ag/AgCl reference electrode and a platinum strip used as a counter electrode. The reaction is as follows:
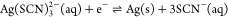
1After the silver electrodeposition process, the network thickness *t* is approximately 24 μm. A schematic view of the sample is presented in Figure 1a.

### 2.2 Ag-NW Network Imaging

**Figure 2:**
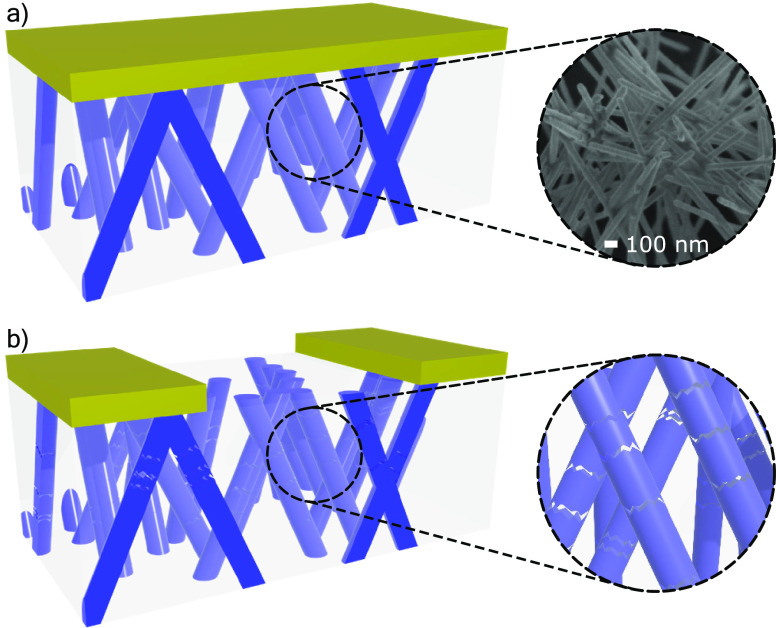
Scheme of the 3D interconnected Ag-NW network and the Au cathode. (a) Before the Au cathode etching, the NW network is intact. The inset shows the SEM image of the Ag-NW network after the dissolution of the membrane. (b) After the Au cathode etching, only two electrodes remain. The inset shows that the etching process has damaged the nanowires. The damages are represented as gaps. The damaged network was not imaged by SEM, as it would collapse once the membrane was removed.

The morphology of the interconnected Ag-NW network was characterized using a field-emission scanning electron microscope (FE-SEM). For the electron microscopy analysis, the PC template was removed by chemical dissolution using dichloromethane from Sigma-Aldrich. As the network density is low, the network collapses once the membrane is completely removed, as shown on the SEM image in Figure 1b. A scheme of the Ag-NWs encapsulated in the membrane with the Au cathode is presented in Figure 2a, the inset of which shows a closer and tilted view of SEM image of the 3D interconnected Ag-NW network. As it can be seen, the Ag-NW network has a complex interconnected structure.

The 3D structure presents a high number of interconnections with the possibility of recurrent connections, with the advantages of NWs with a regular diameter and length. The number of interconnections is estimated numerically as 5.5 × 10^8^ ± 1.0 × 10^8^ interconnections per mm^3^. For the network thickness, the density is around 10^7^ interconnections per mm^2^, which is one order of magnitude greater than a recent structure.^19^ It is interesting to note that the interconnections between Ag NWs share different portions of volume from none (i.e., no crossing) to complete crossing, as shown in Figure 1a.

### 2.3 Etching Process

During the etching process, the cathode is partially removed to create a two-electrode device for electrical measurements.^20^ This process is calibrated with respect to the metallic bilayer thickness. After the complete etching of the cathode between the contact pads, the sample exhibits a ohmic resistance of 7.6 Ω. To trigger memristive behavior, the etching time is further increased.

Although the impact of the plasma etching step on the degradation of the nanowire network is not completely clear, it results in a very noticeable increase in the temperature of the nanocomposite system during this step. As the thermal expansion of the PC membrane^21^ (close to 7 × 10^–5^ K^–1^) is much higher than that of the silver nanowires^22^ (on the order of 10^–5^ K ^–1^), and the volume fraction occupied by the Ag-NWs is on the order of 0.4%, this could result in high internal stresses, leading to ruptures within the NW network and the creation of insulating domains. This hypothesis is supported by the significant increase in resistance (from several Ω to several GΩ) and by the appearance of tunneling at high etching times. The damages are represented as gaps in Figure 2.

This two-step fabrication process allows the production of NWs below the lithography limit with numerous interconnections, a complexity beyond crossbar architecture, and also provides an easy scaling-up of the system by simply increasing its surface. As the Ag-NWs are already interconnected, the membrane is not dissolved at the end of the fabrication. Thus, the network has enhanced solidity, is flexible,^23^ and is protected against oxidation.

### 2.4 Electrical Characterization

The measured sample was about 5 mm long and 1 mm wide, and the electrical contacts were directly made by silver paint. The *I*–*V* curves of the Ag-NWs were obtained using a Keithley 617 electrometer. All measurements are made at room temperature, and tests were made to discard any electrical conduction via the PC membrane to ensure complete conduction through the Ag-NWs and their interconnections only.

## 3 Results and Discussion

### 3.1 High-Resistance Regime

**Figure 3:**
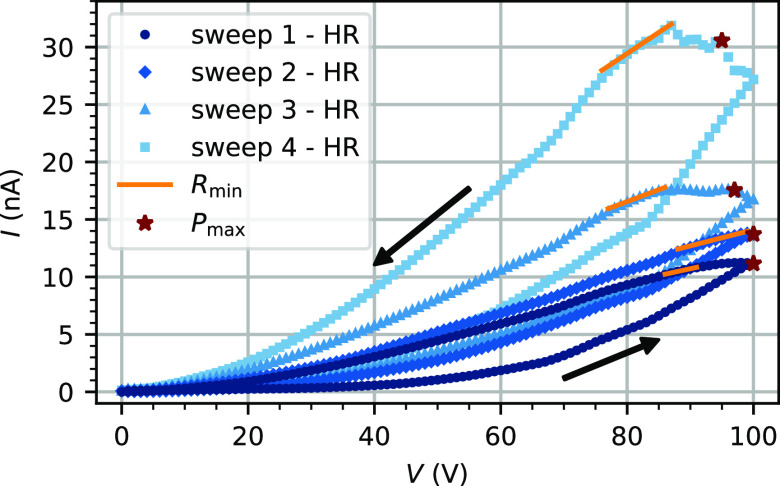
Consecutive *I*–*V* cycles in the high-resistance regime (HR). A triangular voltage ramp is used. It starts at 0 V, increases to 100 V, and finally decreases back to 0 V. The arrows show the evolution of the hysteresis with the voltage. For each curve, the local minimum resistance is represented by a slope (orange line) and the maximal power is indicated by a brown star. Typical memristive behavior can be seen for each consecutive sweep.

An *I*–*V* cycle is repeated several times with a voltage ramp increasing from 0 to 100 V before decreasing back to 0 V with a time step of 1 V/s (see Figure 3). The data are denoised thanks to a digital wavelet transform,^24^ and the same offset is added to each sweep to ensure a positive current. Four consecutive cycles are plotted in Figure 3. For each cycle, there is a local minimum resistance. These values are 8.40 ± 0.02, 7.13 ± 0.06, 4.86 ± 0.04, and 2.73 ± 0.03 GΩ. One can notice that the local minimum resistance decreases with each consecutive cycle. Even with a voltage ramp reaching a high voltage (100 V), as the current flowing in the device is in the nA range, the maximal power reached for each sweep is respectively 1.12, 1.37, 1.70, and 2.90 μW. This device is then resilient to high input voltage with a moderate power consumption.

One can see the typical behavior of a memristive system for all measurements. Similar results were obtain in bulk silver nanowire–polystyrene composites^25^ with an applied voltage ramp up to 160 V and with a PVP-coated Ag-NW network^26^ for a threshold voltage below 2 V. The memristive behavior is expected to arise from the creation of field-induced Ag CF when the voltage increases in the gaps created during the plasma etching. The formation of the CF filaments is volatile, as the conductivity of the sample decreases when the voltage decreases as well. The destruction of some CF filaments might come from thermal effect due to Joule heating or from other phenomena like Rayleigh instability^27,28^ to minimize the system energy or the Gibbs–Thomson effect^29^ due to surface diffusion.

### 3.2 Tunneling Effect

**Figure 4:**
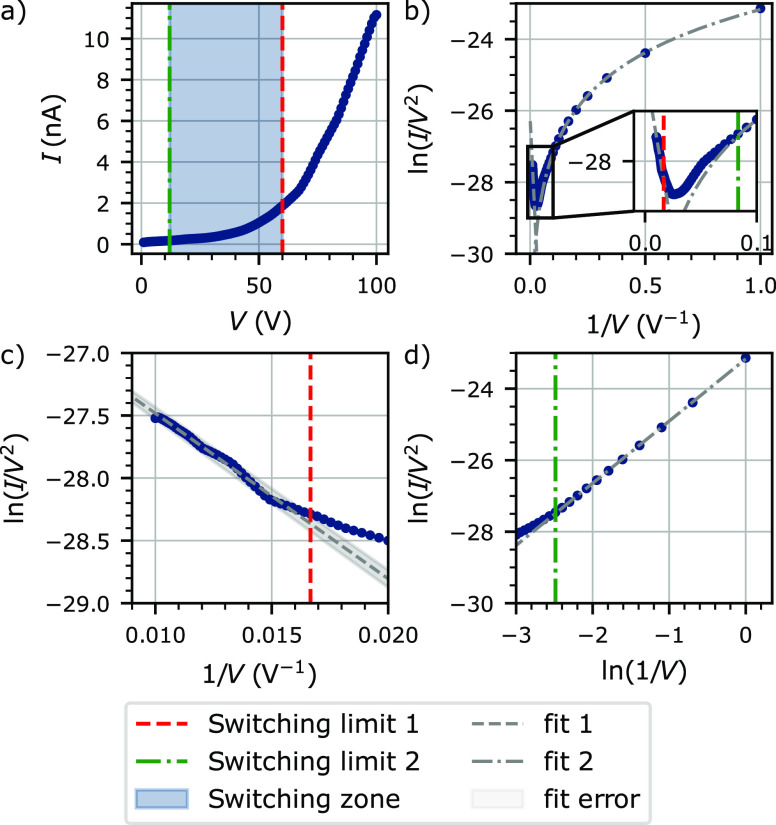
Tunneling phenomena in sweep 1-HR. (a) Data are extracted from sweep 1 until *V* = 100 V. (b) Fowler–Nordheim plot from data presented in (a). The inset shows a zoomed-in view of the switching region. (c) Field-emission at high voltage. (d) Direct tunneling at low voltage. Fits 1 and 2 are linear fitting with *y* = *ax* + *b*, and the fitting error limits are determined using *y* = (*a* ± Δ*a*) *x* + (*b* ± Δ*b*), where Δ*a* and Δ*b* the errors on the parameters *a* and *b* after fitting, respectively. The coefficient of determination *R*^2^ is 0.99 for both fits.

For the first sweep, a change in conduction occurs when a threshold voltage *V*_th_ is exceeded, which leads to a strong current increase. Below this threshold, the increase of the current *I* with the voltage *V* is much smaller. This can be explained by the switching between direct tunneling at low voltage and field emission at high voltage as a Fowler–Nordheim plot suggests^30^ (see Figure 4b). Similar results have been reported with another type of Ag-NW network.^13^ We suppose that the tunnel conduction arises when nanowires are separated by an empty zone or gap acting like an insulating barrier, which appears during the etching of the cathode.

In order to have an estimation of the switching zone between these two regimes, a linear fitting is performed on a selection of data for both field emission and direct tunneling (respectively “fit 1” and “fit 2” in Figure 4). For the field emission, a linear dependence is shown when ln(*I*/*V*^2^) is plotted as a function of 1/*V* as it appears in Figure 4c. The direct tunneling exhibits a linear dependence when ln(*I*/*V*^2^) is plotted as a function of ln(1/*V*), as shown in Figure 4d. An error is extracted from “fit 1” (respectively “fit 2”), and the switching limits are determined as the first (respectively last) value outside the fitting error range. The error of “fit 2” is small and is hardly visible in Figure 4d. These two switching limits are 12 and 60 V for the direct tunneling and field emission, respectively. The switching of the tunneling conduction arises from a change of the tunneling barrier shape from a rectangular barrier to a triangular barrier,^30^ which enhances the probability of tunneling.

### 3.3 Low-Resistance Regime

We measured this sample with *I*–*V* cycles six times before its resistance dropped to the MΩ range and below. This new regime is called the low-resistance regime (LR) due to the decrease of the minimum resistance by a factor of about 47 000. After several repeated *I*–*V* cycles, we suppose that some filaments present an extended lifetime, leading to a strong decrease of the resistance. A similar behavior was reported by Avizienis et al.^10^

**Figure 5:**
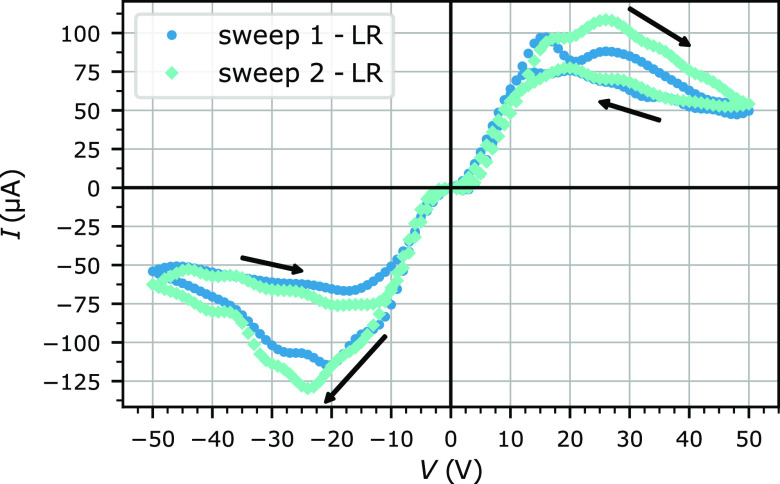
*I* – *V* cycles for two consecutive measurements in the low-resistance regime. The voltage ramp starts at 0 V, increases to 50 V, decreases to −50 V, and finally increases back to 0 V. The arrows show the direction of the current evolution with the voltage ramp.

Several measurements are made in the low-resistance regime, and two consecutive *I*–*V* cycles are reported in Figure 5. The data are denoised in two steps. First, outliers above 200 μA are suppressed and replaced thanks to an interpolation. Then, the data are denoised with a digital wavelet transform.^24^ As seen in Figure 5, different memristive behavior is observed compared to that in the high resistance regime, and a negative differential resistance^31−34^ is observed. Additionally, between +5 and −5 V, diode-like behavior is noticed; further studies with a smaller voltage step would be needed to draw a conclusion on this phenomenon.

The following voltage cycle is applied to the sample: 0 V → 50 V (*V*_stop_) → 0 V → −50 V (−*V*_stop_) → 0 V. First, the current increases with the voltage up to a peak voltage *V*_p1_ of 16 and 26 V for the first and second sweep, respectively. Then, there is a negative differential resistance when the current decreases whereas the voltage increases from *V*_p1_ up to *V*_stop_ and when the current increases whereas the voltage decreases between *V*_stop_ and a second peak voltage *V*_p2_ (14 and 20 V for the first and second sweep, respectively). Finally, from *V*_p2_ to 0 V, the current decreases with the voltage. A symmetrical curve is observed during the second part of the voltage cycle with *V*_p1′_ and *V*_p2′_, which are equal to −20 and −17 V for the first sweep and −24 and −19 V for the second sweep. Similar differential negative resistances were observed in porous silicon^34^ with *V*_p1_ = 7.5 V, *V*_p2_ = 6.0 V, and *V*_stop_ = 20 V. Additionally, multilevel unipolar resistive switching was measured in the Ag/SiO_2_/Pt structure^35^ with *V*_p1_ = −0.6 V and *V*_stop_ between −1 and −4 V.

During the first part of the sweep, a local maximum and two local minima of resistance are reached. The corresponding resistances are given in Table 1 before being averaged. The resulting averaged minimum resistance is *R*_min_^LR^ = 0.163 ± 0.021 MΩ and the averaged maximum resistance is *R*_max_^LR^ = 0.965 ± 0.083 MΩ, leading to a factor of six between the minimum and the maximum averaged resistance in the first-half of the sweeps. Similar results were obtained in the second half of the sweeps with *R*_min_^LR^ = 0.156 ± 0.046 MΩ and *R*_max_^LR^ = 0.862 ± 0.123 MΩ. In this low-resistance regime, the minimum resistances are in the kΩ range, with a voltage threshold in the order of 2 V. The *R*_max_^LR^/*R*_min_^LR^ ratio and the voltage threshold are respectively smaller and larger than those in previous studies.^28,35^ However, the device has an adjustable 3D structure that could be optimized to both increase the *R*_max_^LR^/*R*_min_^LR^ ratio and decrease the voltage threshold by varying the thickness of the network, the NW density, and the NW diameter or by optimizing the etching process.

**Table 1:** Minimum and Maximum Resistances in the Low-Resistance Regime and Their Associated Voltages during the First Half of the Sweeps^a^

	sweep 1-LR	sweep 2-LR
*R*_min,fwd_^LR^	0.150 MΩ at 13 V	0.168 MΩ at 15 V
*R*_max_^LR^	1.007 MΩ at 50 V	0.923 MΩ at 50 V
*R*_min,bwd_^LR^	0.157 MΩ at 10 V	0.178 MΩ at 9 V

a The resistance *R*_min,fwd_^LR^ is measured in the first part of the first sweep when *V* increases from 0 to 50 V, while *R*_min,bwd_^LR^ is measured in the second part when *V* decreases from 50 to 0 V. The maximal resistances were taken at 50 V for clarity.

## 4 Conclusion

In conclusion, we reported a 3D interconnected Ag-NW network with a simple, low-cost, and reliable two-step fabrication process comprised of a template-assisted electrodeposition and an etching process that leads to a highly and randomly interconnected network. The etching process has a double function, as it both damages the Ag NWs, giving rise to memristive properties to the 3D interconnected Ag-NW network, and defines electrode pads for electrical connection. In the future, the etching pattern will be tuned from a basic two-electrode design to a more elaborate pattern with numerous input/output pads. The resulting NWs are highly ordered and encapsulated in a 3D nanoporous polymer film. The 3D architecture allows 5.5 ± 1.0 × 10^8^ interconnections per mm^3^ with an increased complexity, as the interconnections are random with a variety of crossing types. The number of interconnection can be easily adapted by adjusting the NW diameter as well as the membrane porosity. Two resistance regimes are measured with different resistance ranges: in the GΩ range and above for the high-resistance regime and in the MΩ range and below for the low-resistance regime. A tunneling conduction arises in the high-resistance regime, a negative differential resistance is measured in the low-resistance regime, and both regimes exhibit memristive behavior. This device exhibits several complex conduction regimes, and after optimization to decrease the voltage threshold it is anticipated that the low-resistance regime of this device might be useful for memristive applications and even neuromorphic computing,^36,37^ where the high density of interconnections could be exploited to train multiple learning processes on the same device, as numerous pathway are expected to grow in the Ag-NW network.^26^
